# What is the mechanism of paroxysmal atrioventricular block in a patient with recurrent syncope?

**DOI:** 10.1002/joa3.12245

**Published:** 2019-09-27

**Authors:** Raghav Bansal, Ankit Mahajan, Chetan Rathi, Akshay Mehta, Yash Lokhandwala

**Affiliations:** ^1^ Electrophysiology Holy Family Hospital Bandra India; ^2^ Cardiology Brahmakumari Hospital Mumbai India; ^3^ Arrhythmia Associates Mumbai India

**Keywords:** bradycardia, pacemaker, phase 4 block

## Abstract

Paroxysmal atrioventricular (AV) block is characterized by sudden appearance of complete heart block with no escape rhythm. Three types have been described having different mechanisms namely, vagally mediated, intrinsic, and idiopathic. A rare case scenario is being described with the occurrence of paroxysmal AV block of all three types in the same patient.

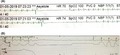

## 1 INTRODUCTION

Paroxysmal atrioventricular (AV) block is an uncommon cause of syncope which can often be missed or overlooked because of its unfamiliarity, unpredictability, and frequent absence of cardiac and resting ECG abnormalities especially with no clear evidence of AV conduction disease. It can have several underlying mechanisms—vagally mediated, intrinsic (Phase 4), and idiopathic. The distinction is important in view of clinical implications.

## 2 CASE REPORT

**Figure 1:**
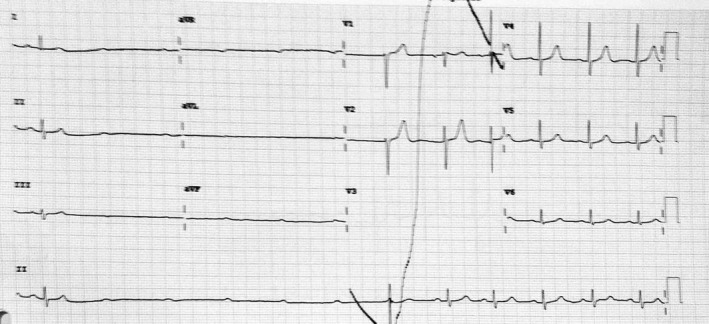
Complete AV block with sinus slowing during carotid sinus massage

**Figure 2:**
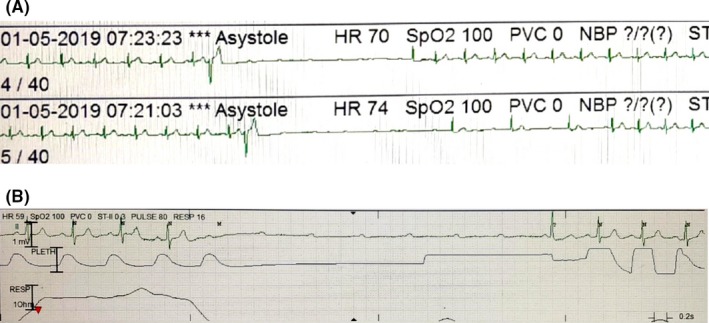
Single‐channel ECG recordings from ICU monitor. A, shows paroxysmal Phase 4 block triggered by a PVC. B, shows idiopathic paroxysmal AV block occurring without any obvious trigger

A 79‐year‐old hypertensive woman presented with recurrent episodes of syncope over a month, increasing in frequency over the last 3 days. Echocardiography revealed fair left ventricular contractility, mild aortic stenosis, marked mitral annular calcification, mild mitral stenosis and mild mitral regurgitation. Her baseline ECG was normal. Carotid sinus massage triggered a paroxysmal AV block with slowing of sinus rate, associated with near‐syncope (Figure 1). During hospital stay, the patient went intermittently into complete AV block (Figure 2), often with near‐syncope. The patient was started on isoprenaline infusion at 2 µg/min, with which the episodes subsided. Subsequently, a dual‐chamber pacemaker was implanted, with alleviation of symptoms. What is the mechanism of paroxysmal AV block in this case?

## 3 COMMENTARY

Rosenbaum et al^1^ defined paroxysmal AV block as an abrupt and unexpected complete AV block in a patient with otherwise 1:1 AV conduction, with delayed ventricular escape. However, there is no consensus over the exact definition of the condition. It is considered an ominous arrhythmia, given its propensity to cause syncope and sudden cardiac death. The prevalence is not known and the condition itself remains under‐recognized as it is often difficult to document. Three major types of paroxysmal AV block have been identified: vagally mediated, intrinsic (Phase 4), and idiopathic.^2^, ^3^

Figure 1 demonstrates the sudden onset of complete AV block during carotid sinus massage, associated with slowing of sinus rate. Resumption of normal AV conduction is accompanied by gradual restoration of sinus rate. This is suggestive of vagally mediated paroxysmal AV block.

Vagally mediated paroxysmal AV block occurs secondary to a surge in parasympathetic activity, is localized within the AV node, associated with a narrow QRS complex escape rhythm, and is relatively benign. It may be associated with identifiable triggers including vomiting, micturition, intense coughing, or phlebotomy. Carotid sinus stimulation, as documented in the current case, causes sympathetic inhibition and a vagal surge. Often, but not always, premonitory symptoms may also occur consisting of diaphoresis, nausea, and dizziness. Although the presence of clinically identifiable triggers and prodromal symptoms suggests a diagnosis of vagal syncope, their absence does not draw a contrary conclusion. It may be asymptomatic, as noticed on Holter recordings, especially during nighttime. ECG recognition is straightforward, with the AV block being preceded by gradual slowing of sinus rate and increase in PR interval. The sinus rate continues to slow down during the ventricular asystole, followed by gradual improvement in AV conduction along with acceleration of sinus rate. The first few beats after resumption of AV conduction usually conduct with a prolonged PR interval.

In Figure 2A, a premature ventricular complex (PVC) triggers a paroxysmal AV block with no associated sinus rate slowing, suggestive of an intrinsic paroxysmal AV block.

Classically described as pause‐dependent Phase 4 AV block, this type of block is associated with a diseased His‐Purkinje system (HPS). Many authors consider only this type of block as paroxysmal AV block and differentiate it from the vagally mediated block.^4^ Phase 4 (pause‐dependent or bradycardia‐dependent) aberrancy or block occurs when the supraventricular impulse is unable to conduct in the diseased HPS.^5^ This is a consequence of partial depolarization in the HPS occurring during the latter part of Phase 4, leading to inactivation of sodium channels, unable to cause a complete depolarization by itself. Intrinsic paroxysmal AV block is triggered by a pause following a PVC, a premature atrial complex, or an abrupt change in heart rate when the impulse reaching the HPS finds it inactive and is unable to conduct. When a critical membrane potential is reached after a critically timed pause, the sodium channel continues to be inactive and unavailable for conduction till the membrane potential is reset to an excitable state by another premature depolarization. This type most commonly occurs in the middle to elderly age group. It has been rarely reported in acute coronary syndromes with inferior or anterior myocardial infarctions. Baseline ECG abnormalities suggestive of conduction system disease may be noted and include right bundle branch block, left bundle branch block, and intra‐ventricular conduction defect in decreasing order. However, in up to one third of the patients, the baseline ECG may be normal.^4^

Figure 2B reveals paroxysmal AV block with no apparent triggers and is termed as idiopathic paroxysmal AV block.

Idiopathic paroxysmal AV block is a relatively novel entity and has features that are atypical and not fitting with the earlier two types. Paroxysmal AV block occurs in the absence of any cardiac or baseline ECG abnormalities, and without any known trigger. The mechanism of idiopathic paroxysmal AV block remains uncertain, with a possibility of a concealed His bundle ectopic/PVC being the trigger. Recently, the role of lower baseline adenosine levels along with excessive susceptibility to exogenous adenosine has also been suggested.

Pacemaker implantation has high efficacy in intrinsic and idiopathic paroxysmal AV block with <5% recurrence of syncope.^2^ A single‐chamber implantation may be considered in idiopathic subtype in view of intermittent requirement of pacing. However, a dual‐chamber pacemaker should be contemplated in intrinsic paroxysmal AV block considering the likelihood of progression of complete AV block on follow‐up as in this case (supplementary figure). Vagal paroxysmal AV block respond inadequately to pacemaker therapy with a higher recurrence rate of syncope (5%‐20%) owing to the presence of associated vasodepressor response.^2^

## 4 CONCLUSION

Paroxysmal AV block is recognized with sudden and unpredictable onset of complete AV block with poor escape rhythm, with otherwise 1:1 AV conduction. It may be vagally mediated, secondary to intrinsic His‐Purkinje disease or idiopathic. Clinical and ECG features may help in differentiating the three types of the block. It is unique, as in our patient, to have all these three mechanisms. It is further emphasized that the presence of one mechanism of paroxysmal AV block does not rule out others. Importantly, the high sensitivity to carotid sinus massage usually indicates a poorer response to pacemaker treatment, but the other findings in this case would support a high effectiveness of pacemaker, as was borne out on follow‐up.

## CONFLICT OF INTERESTS

The authors declare no conflict of interests for this article.
