# Supplementary figures and images for: What is the mechanism of paroxysmal atrioventricular block in a patient with recurrent syncope?
Source: J Arrhythm. 2019 Sep 27;35(6):870–2. doi: 10.1002/joa3.12245 (PMC6898553; doi:10.1002/joa3.12245)

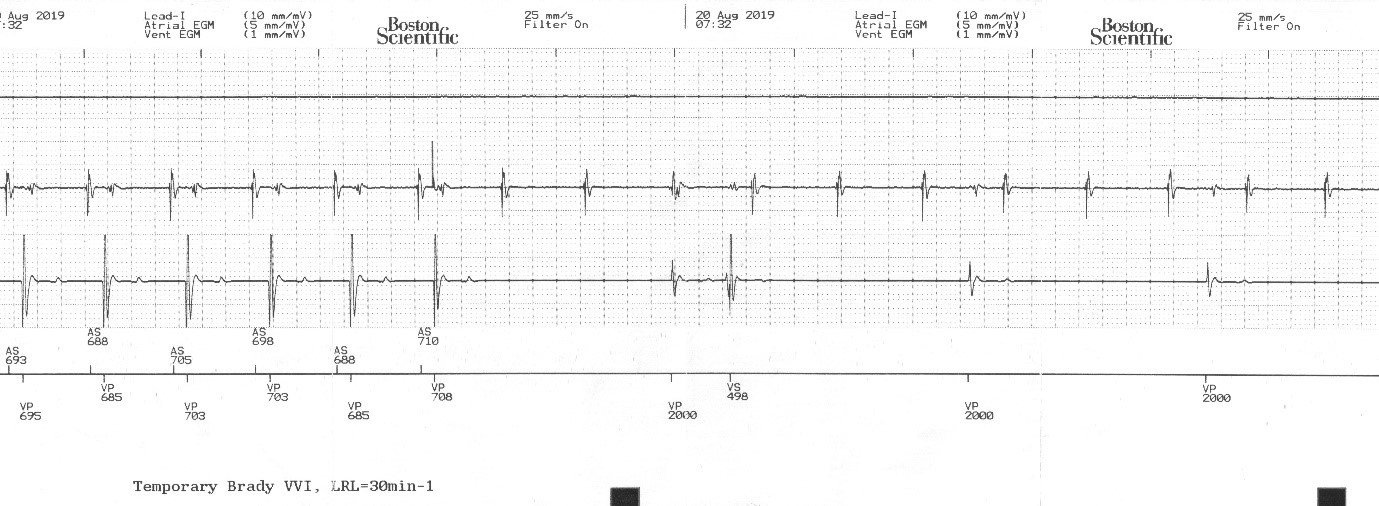

Supplement: Supplementary file 1 [file JOA3-35-870-s001.jpg]
